# Lower Plasma Brain-Derived Neurotrophic Factor Levels During Critical Illness Are Associated with Subsequent Dialysis Requirement

**DOI:** 10.3390/medicina62101842

**Published:** 2026-09-23

**Authors:** Caroline Fromm, Lorena Fröhlich, Veronika Gepperth, Lea Läber, Stephan Schmid, Martina Müller, Vlad Pavel, Patricia Mester, Christa Buechler

**Affiliations:** Department of Internal Medicine I, Gastroenterology, Hepatology, Endocrinology, Rheumatology, and Infectious Diseases, University Hospital Regensburg, 93053 Regensburg, Germany; caroline-anna.fromm@stud.uni-regensburg.de (C.F.); lorena.froehlich@stud.uni-regensburg.de (L.F.); veronika.gepperth@klinik.uni-regensburg.de (V.G.); lea.laeber@klinik.uni-regensburg.de (L.L.); stephan.schmid@klinik.uni-regensburg.de (S.S.); martina.mueller-schilling@klinik.uni-regensburg.de (M.M.); vlad.pavel@klinik.uni-regensburg.de (V.P.); patricia.mester@klinik.uni-regensburg.de (P.M.)

**Keywords:** dialysis, glomerular filtration rate, pancreatitis, sepsis

## Abstract

*Background and Objectives*: Brain-derived neurotrophic factor (BDNF), best known for its role in cognitive function, is an anti-inflammatory protein proposed as a potential therapeutic target and diagnostic biomarker in sepsis. However, studies investigating circulating BDNF levels in patients with sepsis have yielded conflicting results, reporting both increased and decreased concentrations. Comorbid conditions, particularly liver cirrhosis, pancreatitis, and kidney failure, may contribute to these discrepancies. Therefore, the primary aim of this study was to investigate the association between plasma BDNF levels and these comorbidities in patients with sepsis. *Materials and Methods*: Plasma BDNF concentrations were measured in 250 critically ill patients with systemic inflammatory response syndrome (SIRS), sepsis, or septic shock and 40 healthy controls. Among these patients, 56 had liver cirrhosis, 44 had pancreatitis, and 94 required dialysis. *Results*: Patients with SIRS/sepsis/septic shock exhibited significantly lower plasma BDNF levels than healthy controls. Plasma BDNF concentrations were unchanged in patients with concomitant liver cirrhosis, increased in the 44 patients with pancreatitis, and significantly decreased in those with acute kidney injury requiring dialysis (AKI-D), compared with patients without pancreatitis and those without AKI-D, respectively. When patients were stratified by dialysis status, plasma BDNF levels were similar among patients with SIRS, sepsis, and septic shock. Plasma BDNF levels did not change in ventilated patients or in those who did not survive. Furthermore, patients with COVID-19-related sepsis and those with non-COVID-19 etiologies showed comparable plasma BDNF concentrations. However, plasma BDNF levels in non-dialysis and dialysis patients were not, or were only weakly, associated with routine measures of renal function. Plasma BDNF levels positively correlated with platelet count, which predicted BDNF levels in patients with SIRS/sepsis/septic shock. *Conclusions*: Plasma BDNF levels are similarly reduced in patients with SIRS/sepsis/septic shock and are further decreased in AKI-D, whereas patients with pancreatitis exhibit higher BDNF levels. Platelet count, which decreased in AKI-D and was higher in pancreatitis, predicted plasma BDNF levels, suggesting platelet count as an important factor affecting plasma BDNF levels.

## 1. Introduction

Brain-derived neurotrophic factor (BDNF) is essential for the maintenance of neurons in the central and peripheral nervous systems, serving as a general promoter of brain health [1,2,3]. BDNF plays a critical role in cognitive function, and cognitive impairment is a common and clinically significant sequela of sepsis [1,2,3,4].

BDNF also plays an important role in regulating inflammation [1]. It exerts anti-inflammatory effects, at least in part, by binding to toll-like receptor 4 and antagonizing toll-like receptor 4 signaling in macrophages [5]. In a rodent sepsis model, BDNF injection after cecal ligation and puncture reduces tumor necrosis factor levels [6]. Thus, it is appropriate to evaluate systemic BDNF levels in patients with severe inflammatory diseases to elucidate its potential as a biomarker for disease severity.

Infection with SARS-CoV-2 typically causes mild or moderate disease, but it can also lead to sepsis [7]. A pilot study published in 2020 reported lower serum BDNF levels in patients with moderate or severe COVID-19 compared with those with mild disease [8]. Reduced serum BDNF levels in COVID-19 patients compared with controls were also reported in a subsequent study, which showed a further decline in patients with central nervous system manifestations and those requiring ventilation [9]. A study that enrolled 10 male patients with COVID-19 who had died, were in the normal ward, or required intensive care observed similar BDNF levels in these three cohorts, which were all lower compared to healthy controls [10]. However, Ong et al. observed higher plasma BDNF levels early in COVID-19, with no difference between mild, moderate, or severe disease [11]. Elevated concentrations of BDNF were also detected in both saliva and serum during the acute stage of COVID-19. Over the following six months, which corresponded to the remission period, these levels declined but did not return to baseline [12]. However, another study reported that when patients recover from COVID-19, systemic BDNF levels increase [8].

In patients with sepsis of different etiologies, serum BDNF levels were significantly lower than in healthy controls, indicating that reduced BDNF levels are associated with severe disease rather than being specific to SARS-CoV-2 infection [6,9]. Serum BDNF levels of non-survivors were lower than those of survivors at discharge [6]. Plasma BDNF levels at hospital admission were also higher in patients requiring intensive care than in controls. However, a decline on day 2 after hospital admission was associated with higher mortality [13].

Serum and plasma BDNF levels differ greatly. During blood clotting, platelets release BDNF, and serum BDNF levels are higher than plasma levels [14]. Previous studies reported strong positive correlations between plasma and serum BDNF levels, but others could not confirm this [15,16,17]. Therefore, it is important to clearly indicate whether serum or plasma was used for analysis, and the type of anticoagulation may also affect BDNF levels in the blood samples [14,18]. Current data on systemic BDNF levels in severe infectious diseases such as COVID-19 remain discordant, and clarifying the underlying reasons—particularly the contribution of coexisting organ dysfunction—was a central motivation for the present study.

BDNF is not exclusively expressed in the brain; it is also produced in peripheral tissues, including skeletal muscle, kidney, and the liver [1,19], suggesting that underlying diseases may affect its systemic levels. BDNF is expressed in hepatocytes and hepatic stellate cells, where it exerts protective effects against hepatic inflammation and fibrosis [19,20]. Supporting an association of BDNF with liver function, mice with diminished BDNF expression develop obesity, insulin resistance, and non-alcoholic steatohepatitis [21]. In obese mice, BDNF administration improved blood glucose regulation and liver function [22]. These observations suggest that circulating BDNF levels may be reduced in individuals with metabolic dysfunction and/or chronic liver disease. However, elevated serum BDNF concentrations have been reported in patients with obesity and type 2 diabetes, with positive associations between BDNF and fat mass, fasting glucose, insulin resistance, and triglyceride levels [23,24]. These observations align with data showing that hepatic BDNF contributes to insulin resistance and liver disease, as shown in liver-specific BDNF-null mice [25]. Notably, BDNF secreted by skeletal muscle cells enhances glucose-stimulated insulin secretion by pancreatic β-cells, whereas BDNF derived from the pancreas or liver does not, indicating that the tissue of origin influences the biological activity of BDNF [25,26].

Liver cirrhosis is a risk factor for sepsis severity, and acute -on-chronic liver failure is a life-threatening complication in sepsis [27]. However, clinical findings on blood BDNF levels in cirrhosis are inconsistent. Female patients with liver cirrhosis exhibited higher serum BDNF levels than healthy controls [28]. In contrast, several other studies reported significantly lower serum BDNF concentrations in cirrhotic patients than in controls [29,30,31]. Thus, underlying liver cirrhosis in sepsis may be one confounder contributing to the discordant results in previous studies analyzing blood BDNF levels of patients with sepsis [9,12]. Moreover, chronic kidney disease has been associated with low systemic BDNF levels [32], but whether BDNF levels also decrease in patients with sepsis requiring dialysis has not been evaluated to our knowledge.

Therefore, this study aimed to evaluate the association of liver cirrhosis and/or renal failure with circulating BDNF levels in patients with sepsis. In addition, we investigated associations with pancreatitis, severity of critical illness, need for ventilation, and survival, and compared COVID-19–related and non-COVID-19 sepsis.

## 2. Materials and Methods

### 2.1. Patients and Controls

Plasma specimens were collected from 250 individuals admitted to the intensive care unit (ICU) of the University Hospital of Regensburg between September 2018 and February 2026. Individuals with documented hepatitis virus infection or human immunodeficiency virus infection were not eligible for inclusion. The study population comprised 57 patients with systemic inflammatory response syndrome (SIRS), 66 with sepsis, and 127 with septic shock. Among the sepsis and septic shock cases, 29 patients were hospitalized due to SARS-CoV-2 infection. Samples from COVID-19 patients were obtained between October 2020 and October 2024. Patients were classified as having SIRS, sepsis, or septic shock based on SIRS criteria and the Sepsis-3 definitions [33,34]. Clinically, SIRS is described by the presence of two or more symptoms, including fever or hypothermia, tachycardia, tachypnoea, and pathological change in blood leucocyte count. According to the Sepsis-3 definition [33], sepsis was diagnosed in patients with an acute increase of ≥2 points in the SOFA score in the presence of a suspected or confirmed infection. Furthermore, septic shock cases required vasopressor therapy to maintain a mean arterial pressure ≥ 65 mmHg and an increase in lactate concentration greater than 18 mg/dL. Infection was confirmed by microbiological investigations or, in cases of negative microbiological results, suspected based on clinical or radiological features. All patients with SIRS had a proven or suspected infectious cause.

The median SOFA score at admission was 9 (range, 5–13) in the sepsis group and 19 (range, 15–23) in the septic shock group. The median APACHE II score was 25 (range, 14–31) for patients with sepsis and 56 (range, 40–69) for those with septic shock.

At the time of sample collection, median lactate was 22 mg/dL (8–33 mg/dL) in the sepsis cohort and 56 mg/dL (23–167 mg/dL) in the septic shock cohort. At the time of blood sample collection, patients experiencing septic shock were receiving median doses of 0.6 µg/kg/min of norepinephrine and 0.05 units/min of vasopressin.

In patients undergoing invasive mechanical ventilation, the median PaO_2_/FiO_2_ ratio (Horowitz index) was 138 mmHg (range, 56–234). For vasopressor therapy and mechanical ventilation, we obtained samples after initiating these interventions.

For patients on dialysis, we collected blood samples before dialysis. None of the patients were on dialysis before ICU admission. Creatinine, estimated glomerular filtration rate (eGFR), and urea at admission are given in the manuscript. Patients who required dialysis developed renal failure stage 3 according to the Kidney Disease: Improving Global Outcomes (KDIGO) classification. The indication for renal replacement therapy (RRT) was septic acute renal failure with oligo-anuria. All patients received continuous veno-venous hemodialysis. While BDNF sampling was performed within 24 h after ICU admission, renal replacement therapy was initiated a median of 3 days after admission.

This study prospectively enrolled all ICU patients who consented to participate and met the predefined diagnostic criteria. Patients’ plasma was prospectively collected within 24 h after ICU admission.

Patients discharged alive from the ICU were classified as survivors, whereas those who died during their ICU stay were categorized as non-survivors.

Clinical laboratory data were supplied by the Institute of Clinical Chemistry and Laboratory Medicine.

The study included 40 controls, who were similar in age to the patients (*p* = 0.127) but had a higher proportion of females (*p* = 0.009).

### 2.2. BDNF Measurement with Enzyme-Linked Immunosorbent Assay (ELISA)

The anticoagulant used was ethylenediaminetetraacetic acid (EDTA). The time from blood collection to centrifugation was a median of 3 h. Storage of EDTA plasma for 2 h at room temperature does not affect BDNF levels [14], but whether longer storage time has a significant effect has not been analyzed so far. Centrifugation was performed at 3000× *g* rounds/minute (1620× *g*) to obtain plasma. Plasma specimens were aliquoted and stored at −80 °C until required for analysis. Samples were thawed prior to testing. Samples with one, two, or three freeze/thaw cycles were used, and a previous study showed that two freeze/thaw cycles do not considerably change EDTA-plasma BDNF levels [14]. However, long storage time (1 year) at −80 °C may increase BDNF levels in blood [18]. The median BDNF levels of samples collected early during the study and of the samples collected more recently were, however, similar (median of 2145 and 2466 pg/mL, respectively).

BDNF concentrations were retrospectively determined using the human/mouse BDNF DuoSet ELISA kit (Catalog No. DY248; Bio-Techne, Wiesbaden, Germany) after diluting the plasma 1:50. This kit included 15 plates, which were sufficient to measure all of the samples. Calibration standards and participant samples were measured in duplicate, and the mean of the two replicate measurements was used for data analysis. The inter-assay coefficient of variation (CV) (9 samples measured on two different plates was 13.14%, and the intra-assay CV (30 samples measured in duplicate) was 7.90%. The standard curve was 6.1, 15.4, 38.4, 96.0, 240.0, 600.0, and 1500 pg/mL BDNF.

The Limit of Quantification (LOQ) was the lowest concentration in the calibration curve that demonstrated reliable inter-assay precision (CV < 20%), which was 15.4 pg/mL BDNF. Five plasma samples at a 1:50 dilution had BDNF concentrations below 15.4 pg/mL (6.4–14.3 pg/mL), but the optical density of the lowest standard (6.1) was higher than that of the blank. We used the BDNF concentrations from the ELISA for these samples in our analysis.

### 2.3. Determination of the Glomerular Filtration Rate

The estimated glomerular filtration rate (eGFR) was estimated using the Chronic Kidney Disease Epidemiology Collaboration (CKD-EPI) equations as reported by Inker et al. [35]. The equations are: 144 × (serum creatinine/0.7)^−0.329^ × 0.993^Age^ for females with creatinine ≤ 0.7 mg/dL, 144 × (serum creatinine/0.7)^−1.209^ × 0.993^Age^ for females with creatinine > 0.7 mg/dL, 141 × (serum creatinine/0.9)^−0.411^ × 0.993^Age^ for males with serum creatinine ≤ 0.9 mg/dL, and 141 × (serum creatinine/0.9)^−1.209^ × 0.993^Age^ for males with serum creatinine > 0.9 mg/dL. The use of eGFR is a limitation of our study, as GFR-estimating equations perform less well in critically ill patients than creatinine-based equations in outpatient cohorts [36].

### 2.4. Statistics

Plasma BDNF levels showed a non-normal distribution according to the Shapiro–Wilk test (*p* < 0.001). Therefore, results are displayed as box-and-whisker plots, with extreme values represented separately as points or asterisks. Descriptive statistics are reported as medians together with the corresponding ranges (minimum–maximum values). Hodges-Lehmann median differences with 95% confidence intervals (CIs), 95% CIs, odds ratios, and interquartile ranges (IQRs) were reported where appropriate.

Owing to the non-normal distribution of the data, non-parametric statistical procedures were applied. Differences between two independent groups were assessed using the Mann–Whitney U test, whereas comparisons involving more than two independent groups were conducted with the Kruskal–Wallis test (SPSS Statistics version 31.0; IBM Corp., Armonk, NY, USA).

Multiple linear regression was used to evaluate factors predicting plasma log_10_-transformed BDNF levels. Age, albumin, eGFR, and platelet count were not/weakly correlated (r < 0.152, *p* ≥ 0.016). Missing data were excluded listwise. Homoscedasticity was checked in SPSS using standardized residuals and standardized predicted values. A normal P-P plot was done, and Cook’s distance values were far below 1. There were no standardized residuals falling outside the range of −3.0 and +3.0.

Binary logistic regression was used to predict the need for dialysis using eGFR, creatinine, or BDNF as variables. Receiver operating characteristic (ROC) curve analyses were performed to assess the diagnostic performance of the individual variables. Relationships between variables were evaluated by calculating Spearman’s rank correlation coefficient using SPSS Statistics. Statistical significance was established at a threshold of *p* < 0.05. Categorical variables, such as sex distribution and the frequency of dialysis, were compared using the chi-square test.

We did not adjust the results for multiple comparisons for reasons discussed elsewhere [37,38]. The analyses were specified a priori based on the hypothesis that BDNF levels in patients with SIRS/sepsis/septic shock may be associated with underlying diseases. Consequently, the study was not intended to identify significant associations from a large collection of candidate variables or outcomes. We therefore considered adjustment for multiple comparisons inappropriate as a post hoc statistical penalty for testing a small number of prespecified, clinically related hypotheses. We regarded this analysis as mostly exploratory, and reported data without adjustment for multiple testing.

## 3. Results

### 3.1. BDNF Levels in the Plasma of Controls and Patients with SIRS/Sepsis/Septic Shock

Plasma BDNF levels were measured in 40 controls and 250 patients with SIRS/sepsis/septic shock. In this cohort, plasma BDNF levels did not differ between male and female patients (*p* = 0.466), and the difference between male and female controls did not reach the predefined significance threshold (*p* = 0.050). Both sexes were therefore analyzed together.

Plasma BDNF levels in patients were significantly lower than in controls (patients: 2.4 (0.3–42.8) ng/mL; controls: 5.0 (1.5–24.6) ng/mL; Hodges-Lehmann median difference 1.9; 95% confidence interval (CI) 1.1–2.8; Figure 1a). The control group had more females than males, compared with the patient cohort (*p* = 0.009), and we also conducted sex-specific analyses. Among the 68 female patients, plasma BDNF levels were lower in SIRS/sepsis/septic shock than in the 19 controls (Hodges-Lehmann median difference 2.9; 95% CI 1.1–4.7, *p* = 0.003). Among the 182 male patients, plasma BDNF levels were also reduced compared with the 21 controls (Hodges-Lehmann median difference 1.2; 95% CI 0.27–2.01, *p* = 0.011).

However, when stratified by disease severity, the 57 patients with SIRS, the 66 with sepsis, and the 127 with septic shock had similar plasma BDNF levels (*p* = 0.126; Figure 1b). Accordingly, the 65 non-survivors had plasma BDNF levels comparable to those of the surviving patients (*p* = 0.139; Figure 1c).

SARS-CoV-2 infection can also cause sepsis, which is mostly similar to sepsis of other etiologies [39]. Indeed, the 29 patients with SARS-CoV-2 sepsis had plasma BDNF levels similar to those of patients with other disease etiologies (*p* = 0.305).

### 3.2. BDNF Levels, Underlying Disease, and Interventions

Underlying diseases such as liver cirrhosis may be associated with changed BDNF levels independent of sepsis [30]. Moreover, chronic pancreatitis has been associated with higher BDNF expression [40]. However, plasma BDNF levels in the 56 patients with liver cirrhosis were comparable to those in patients without this condition (*p* = 0.433) (Figure 2a). Notably, platelet count was lower in cirrhosis (*p* < 0.001; Hodges-Lehmann median difference 68; 95% CI 39–106). Pancreatitis in 44 patients was related to higher plasma BDNF levels (Hodges--Lehmann median difference 0.9; 95% CI 0.3–1.8; *p* = 0.004; Figure 2b).

Patients with pancreatitis were younger (*p* = 0.002), had more eosinophils (*p* = 0.006), lymphocytes (*p* = 0.021), and platelets (*p* < 0.001), and had a higher estimated glomerular filtration rate (eGFR) (*p* = 0.023) and gamma-glutamyltransferase (*p* < 0.001) than patients without this underlying disease. BDNF (*p* = 0.018, regression coefficient B: 0.000, odds ratio: 1.00, 95% CI 1.00–1.00), age (*p* = 0.004; regression coefficient B: −0.036, odds ratio: 1.04, 95% CI 1.01–1.06), sex (*p* = 0.028; regression coefficient; B: −1.335, odds ratio: 3.80, 95% CI 1.15–12.50), gamma-glutamyltransferase (*p* = 0.003; regression coefficient B: 0.002. odds ratio: 1.00, 95% CI 1.00–1.00), and platelet count (*p* < 0.001; regression coefficient B: 0.006, odds ratio: 1.01, 95% CI 1.00–1.01) significantly predicted pancreatitis (*p* < 0.001).

Interventions such as ventilation in 164 patients and vasopressor therapy in 179 patients were not associated with changes in plasma BDNF levels (*p* = 0.694 and *p* = 0.135, respectively). These interventions began before plasma collection, whereas renal replacement therapy started after blood collection.

Renal disease is associated with low systemic BDNF levels [32], and the 94 SIRS/sepsis/septic shock patients with acute kidney injury requiring dialysis (AKI-D) had significantly lower plasma BDNF levels than those without (Hodges-Lehmann median difference 0.7; 95% CI 0.3–1.2, *p* = 0.001; Figure 2c).

Patients with pancreatitis who needed dialysis (12 patients, *p* = 0.067) tended to have lower BDNF levels than those who did not require dialysis. Notably, the need for dialysis among patients with pancreatitis was similar to that among patients without pancreatitis (*p* = 0.127). Importantly, the association between pancreatitis and higher plasma BDNF levels was equally evident in the subgroup of patients not requiring dialysis (Hodges-Lehmann median difference 1.2; 95% CI 0.2–3.0, *p* = 0.018), showing that factors beyond dialysis need affect systemic BDNF levels.

Binary logistic regression showed that, in the entire patient cohort, eGFR and creatinine correctly classified dialysis (*p* < 0.001) in 71.2% and 69.1% of patients, respectively (odds ratio for eGFR = 1.03, 95% CI 1.02–1.04; odds ratio for creatinine = 1.77, 95% CI 1.41–2.22). The baseline model, which included none of these predictors, correctly classified 62.4%.

Binary logistic regression including eGFR and BDNF correctly classified 70.7% of patients (odds ratio for BDNF = 1.00, 95% CI 1.00–1.00; odds ratio for eGFR = 1.02, 95% CI 1.02–1.03) and creatinine and BDNF correctly classified 71.3% of patients (odds ratio for BDNF = 1.00, 95% CI 1.00–1.00; odds ratio for creatinine = 1.73, 95% CI 1.38–2.17) showing that including BDNF does not improve dialysis prediction.

The area under the receiver operating characteristic curve (AUROC) for AKI-D prediction in the entire cohorts was 0.739 ± 0.034 for eGFR (95% CI 0.672–0.806), 0.738 for creatinine (95% CI 0.668–0.807) and 0.638 ± 0.037 for BDNF (95% CI 0.565–0.712) (all *p* < 0.001), indicating low to median discriminatory ability and suggesting that neither biomarker has good diagnostic value.

AKI-D patients had higher procalcitonin, aspartate aminotransferase, bilirubin, urea, and creatinine levels, and a lower platelet count and eGFR than those not requiring dialysis (Table 1). Mortality was significantly higher in patients requiring future dialysis (48 of 94; 51%) than in patients not requiring dialysis (17 of 156; 11%; *p* < 0.001), and AKI-D more frequently presented with sepsis and/or septic shock (*p* < 0.001) (Table 1).

AKI-D patients more often had septic shock than non-AKI-D patients (Table 1), and BDNF levels of this subcohort were analyzed separately. In patients with septic shock, 75 patients were on dialysis. They had lower BDNF (Hodges-Lehmann median difference 1.0; 95% CI 0.2–2.1, *p* = 0.008), higher procalcitonin (Hodges-Lehmann median difference 1.2; 95% CI 0.23–2.63, *p* = 0.007), bilirubin (Hodges-Lehmann median difference 1.3; 95% CI 0.5–2.6, *p* < 0.001), creatinine (Hodges-Lehmann median difference 0.73; 95% CI 0.31–1.19, *p* < 0.001), and AST (Hodges– Lehmann median difference 17; 95% CI 2–38, *p* = 0.027), and lower platelets (Hodges--Lehmann median difference 957; 95% CI 243–2193, *p* = 0.013) and eGFR (Hodges-Lehmann median difference 20; 95% CI 8–33, *p* < 0.001) than septic shock patients who did not require dialysis. This subgroup analysis could not clarify whether disease severity is the main cause of low BDNF in AKI-D.

To address this further, we performed multiple linear regression analysis in the entire cohort. Because BDNF levels vary widely in plasma, we used log_10_-transformed BDNF for multiple linear regression analysis. Multiple linear regression including age, sex, underlying liver cirrhosis and pancreatitis, interventions (dialysis, vasopressor therapy and ventilation), disease severity (SIRS/sepsis/septic shock), and eGFR, as well as albumin to represent hepatic synthetic function and platelet count as a source of plasma BDNF levels, revealed a significant prediction of log_10_-transformed plasma BDNF (F(11,207) = 3.621; *p* < 0.001). Here, platelets (*p* = 0.008; regression coefficient B: 0.001) and dialysis (*p* = 0.008; regression coefficient B: −0.173) were significant.

Notably, platelet count differed between patients with SIRS, sepsis and septic shock (*p* = 0.016), with lower counts in septic shock.

### 3.3. BDNF Levels in Patients Not Requiring Dialysis

Because BDNF levels differed substantially between patients with and without AKI-D (Figure 2c), we also analyzed these cohorts separately.

After excluding patients requiring dialysis, the difference in plasma BDNF levels between patients with SIRS/sepsis/septic shock and controls remained significant, with lower BDNF levels in the patient group. SIRS/sepsis/septic shock patients without dialysis had 2.7 (0.4–42.8) ng/mL, and controls had 5.0 (1.5–24.6) ng/mL (Hodges-Lehmann median difference 1.5; 95% CI 0.5–2.5, *p* = 0.003; Figure 3a).

In patients without AKI-D, plasma levels among the 50 patients with SIRS, 54 with sepsis, and 52 with septic shock were similar (*p* = 0.788). The 17 non-survivors did not have plasma BDNF levels that differed from those of survivors (*p* = 0.301).

Liver cirrhosis (29 patients, *p* = 0.786) did not change plasma BDNF levels, which were higher in patients with pancreatitis (32 patients, Hodges-Lehmann median difference 1.2; 95% CI 0.2–3.0, *p* = 0.018). Patients with pancreatitis were younger (Hodges-Lehmann median difference 11; 95% CI 4–17, *p* = 0.004), had more eosinophils (Hodges-Lehmann median difference 0.08; 95% CI 0.02–0.17, *p* < 0.001) and lymphocytes (Hodges-Lehmann median difference 0.33; 95% CI 0.09–0.56, *p* = 0.007), and had higher eGFR (Hodges-Lehmann median difference 23; 95% CI 6–39, *p* = 0.007) and gamma-glutamyltransferase (Hodges-Lehmann median difference 91; 95% CI 46–137, *p* < 0.001) than patients without this underlying disease.

Plasma BDNF levels were not significantly changed by the interventions applied, with comparable values in the 83 ventilated patients (*p* = 0.090) and the 92 patients receiving vasopressors (*p* = 0.821).

COVID-19 patients (18 patients) had plasma BDNF levels similar to those with other disease etiologies (*p* = 0.174).

The association of plasma BDNF levels with renal function parameters was also analyzed. Patients with creatinine levels below 1 mg/dL (61 patients) had higher plasma BDNF levels than patients with creatinine above 1 mg/dL (81 patients; Hodges-Lehmann median difference 0.9; 95% CI 0.1–2.2; *p* = 0.023) (Figure 3b). However, those with urea <50 mg/dL (62 patients) and those with urea >50 mg/dL (94 patients) had similar BDNF levels (*p* = 0.115). Plasma BDNF levels were not associated with eGFR when patients were stratified for eGFR: 60–>90 mL/min/1.73 m^2^ (73 patients), 30–59 mL/min/1.73 m^2^ (44 patients), and 29 to <15 mL/min/1.73 m^2^ (28 patients; *p* = 0.109) (Figure 3c).

### 3.4. BDNF Levels in Patients Requiring Dialysis

Patients requiring dialysis had very low plasma BDNF levels, with a median of 2.0 (0.3–27.3) ng/mL (Hodges-Lehmann median difference 2.4; 95% CI 1.6–3.5, *p* < 0.001 compared to controls; Figure 4a). Excluding patients with very low BDNF levels below the limit of quantification, the median value was 2.0 (1.0–27.3) ng/mL (Hodges-Lehmann median difference 2.3; 95% CI 1.5–3.5, *p* < 0.001 compared to controls).

In patients requiring dialysis, plasma BDNF levels were similar among the 7 patients with SIRS, 12 with sepsis, and 75 with septic shock (*p* = 0.185) and did not differ between survivors and non-survivors (48 patients, *p* = 0.386).

Patients with creatinine below 1 mg/dL (10 patients) had comparable plasma BDNF levels to patients with creatinine above 1 mg/dL (71 patients; *p* = 0.214) (Figure 4b). Those with lower urea (19 patients) and those with urea greater than 50 mg/dL (75 patients) had similar BDNF levels (*p* = 0.194). Plasma BDNF levels were not associated with eGFR when patients were stratified for eGFR: 60–>90 mL/min/1.73 m^2^ (18 patients), 30–59 mL/min/1.73 m^2^ (19 patients), and 29 to <15 mL/min/1.73 m^2^ (47 patients; *p* = 0.101) (Figure 4c). This did not change after excluding patients with very low BDNF levels (creatinine < and > 1mg/dL; *p* = 0.107, urea < and > 50 mg/dL; *p* = 0.083, eGFR 60–>90 mL/min/1.73 m^2^, 30–59 mL/min/1.73 m^2^, and 29 to <15 mL/min/1.73 m^2^ *p* = 0.419).

No difference in plasma BDNF levels was detected in patients with vasopressor therapy (87 patients, *p* = 0.914) or ventilation (81 patients, *p* = 0.865). This also applied to underlying diseases, with 27 patients having cirrhosis (*p* = 0.688) and 12 having pancreatitis (*p* = 0.308). The 11 COVID-19 patients had BDNF levels similar to those of patients with other disease etiologies (*p* = 0.986).

### 3.5. Correlation and Linear Regression Analysis

Correlation analysis was performed separately in patients with and without dialysis (Table 2). In patients without dialysis, plasma BDNF showed a weak positive association with body mass index and weak negative correlations with urea, creatinine, and bilirubin. In patients with dialysis, plasma BDNF showed a weak negative correlation with eosinophil count and a weak positive correlation with urea. All correlation coefficients were low in magnitude and had high *p*-values. Plasma BDNF negatively correlated with direct and indirect bilirubin in non-AKI-D patients and positively correlated with platelet counts in both cohorts, and these associations were highly significant (Table 2).

The associations of plasma BDNF with these measures did not greatly change in AKI-D after excluding patients with very low BDNF levels (eosinophils r = −0.209, *p* = 0.049, 95% CI −0.405–0.005; platelets: r = 0.315, *p* = 0.002, 95% CI 0.110–0.495; urea: r = 0.279, *p* = 0.009, 95% CI 0.066–0.468).

Multiple linear regression including age, sex, underlying liver cirrhosis and pancreatitis, interventions (vasopressor therapy and ventilation), disease severity (SIRS/sepsis/septic shock), and eGFR, as well as albumin to represent hepatic synthetic function and platelet count as a source of plasma BDNF levels, was significant for predicting log-transformed BDNF in non-dialysis patients (*p* = 0.032; F (10,126) = 2.070). Here, platelet count was significant (*p* = 0.017, regression coefficient B: 0.001). This was not significant in AKI-D (*p* = 0.159).

## 4. Discussion

This study demonstrates that SIRS/sepsis/septic shock are associated with reduced plasma BDNF levels, which are even lower in patients with AKI-D. In contrast, pancreatitis was associated with higher plasma BDNF levels. Plasma BDNF levels were largely unrelated to routine renal function parameters but were associated with platelet counts, supporting the notion that platelets may be a major contributor to changes in circulating BDNF levels.

To date, studies investigating circulating BDNF levels in sepsis have yielded inconsistent results [6,9,10]. In our comparatively large cohort, plasma BDNF levels were significantly lower in patients with SIRS/sepsis/septic shock than in healthy controls. These findings are consistent with several previous studies [6,9,10] but differ from others [13].

Although one previous study reported an association between serum BDNF and disease severity in patients with COVID-19 [8], another found no differences in serum BDNF levels across disease severity categories [10], consistent with our findings. Thrombocytopenia is common in critically ill patients, particularly those with septic shock, and is multifactorial in origin [41]. In our cohort, patients with septic shock had lower platelet counts; however, the difference was small, which may partly explain why plasma BDNF levels did not differ between SIRS, sepsis, and septic shock.

Because our center specializes in gastrointestinal diseases, patients with pancreatitis were well represented in our cohort. Pancreatitis was associated with higher plasma BDNF levels, which may reflect the increased BDNF expression reported in pancreatic tissue from these patients [40]. Patients with pancreatitis required dialysis at a frequency similar to patients without pancreatitis, and those undergoing dialysis showed a trend toward lower plasma BDNF levels. These findings suggest that the decline in plasma BDNF associated with dialysis need is preserved despite the increase in BDNF associated with pancreatitis. Patients with pancreatitis had higher platelet counts. In patients with acute pancreatitis, higher platelet counts are associated with disease complications and infections [42]. Although the severity of pancreatitis was not assessed in our cohort, the higher platelet count observed in these patients may not be unexpected and could contribute to the higher plasma BDNF levels, given the substantial contribution of platelets to circulating BDNF [43].

Liver cirrhosis is a recognized risk factor for sepsis and is associated with altered circulating protein levels, including C-reactive protein [44,45]. Previous studies have reported reduced circulating BDNF concentrations in patients with virus-associated liver cirrhosis. For example, patients with hepatitis C virus-related cirrhosis exhibited significantly lower serum BDNF levels than healthy controls [31], and similar findings have been reported in hepatitis B virus-associated cirrhosis [30]. In contrast, plasma BDNF levels were comparable between SIRS/sepsis/septic shock patients with and without liver cirrhosis in our cohort. These findings suggest that chronic virus-induced inflammation, rather than hepatic dysfunction itself, may contribute to the reduction in circulating BDNF observed in virus-associated cirrhosis. This interpretation is supported by the observation that serum BDNF levels increase following successful eradication of hepatitis C virus [46], a setting in which hepatic inflammation improves despite persistent cirrhosis [47,48]. Platelet counts were, however, reduced in patients with SIRS, sepsis, or septic shock and liver cirrhosis. Nevertheless, plasma BDNF levels did not decline in these patients, and the reasons remain unclear.

Notably, patients with AKI-D had lower plasma BDNF levels than patients with SIRS/sepsis/septic shock who did not require renal replacement therapy. Reduced circulating BDNF levels have been reported in patients with chronic kidney disease [32]. Zoladz et al. reported lower serum BDNF levels in patients with chronic kidney disease, which further declined transiently during dialysis, while this effect was not observed in plasma [49]. Postmortem analyses have demonstrated lower BDNF levels in brain tissue from patients with kidney disease than in individuals without kidney disease [50]. Because directly assessing central BDNF in humans is challenging, peripheral BDNF is often used as a surrogate marker. Experimental studies support this approach by demonstrating bidirectional transport of BDNF across the blood–brain barrier [51] and an association between peripheral and central BDNF levels [52]. Approximately 99% of peripheral BDNF is stored in platelets, whereas only a small fraction circulates freely in plasma [53]. As platelets do not cross the blood–brain barrier [54], free plasma BDNF may more closely reflect central BDNF than platelet-associated BDNF. Nevertheless, it remains unclear whether the reduction in brain BDNF observed in kidney disease [50] contributes to the lower plasma BDNF levels in these patients.

Linear regression including age, sex, underlying liver cirrhosis and pancreatitis, interventions, disease severity, creatinine, eGFR and albumin to represent hepatic synthetic function and platelet count as a source of plasma BDNF levels revealed that AKI-D and platelet count were significant predictors in the entire patient cohort. BDNF was positively correlated with platelet counts, and lower platelet counts in AKI-D patients seem to contribute to reduced plasma BDNF levels.

However, CKD-EPI equations with creatinine to assess eGFR in ICU patients perform poorly, partly because critical illness can alter creatinine [36]. In our cohort, plasma BDNF levels were not or only weakly associated with eGFR or creatinine, whose levels are also affected by non-renal complications such as muscle cell loss in ICU patients [55]. Thus, our study does not provide evidence that renal dysfunction leads to lower plasma BDNF in critically ill patients.

In a murine model of unilateral ureteral obstruction, renal BDNF expression was reduced in proximal tubule cells, podocytes, and macrophages, and circulating BDNF levels were low [32]. This suggests reduced renal synthesis as the cause of circulating depletion. BDNF is detected in urine [38], but whether higher urinary excretion contributes to low systemic BDNF in patients with renal disease warrants further study.

This study used EDTA-plasma for BDNF analysis. During blood clotting, BDNF is released by platelets, and serum BDNF levels are higher in comparison to its levels in plasma [14]. EDTA prevents coagulation but does not completely prevent platelet degranulation [56]. Thus, release of BDNF by platelets in our EDTA-plasma samples cannot be completely excluded.

Several limitations of this study should be acknowledged. Factors known to influence serum BDNF concentrations, including cognitive performance and levels of physical activity, were not evaluated [57,58]. A cognitive assessment to corroborate the neurocognitive association of BDNF was not performed during or following the intensive care period. Furthermore, participants were recruited exclusively from a single center in Germany, which may limit the applicability of the results to broader populations with different demographic or ethnic backgrounds. Another limitation is that plasma BDNF measurements were obtained only during the early phase after hospital admission, with no subsequent longitudinal assessments. Correlations of plasma BDNF with the SOFA score, which uses platelet counts, were not analyzed because of similar BDNF levels in patients with SIRS, sepsis, and septic shock, who had greatly different disease scores. In critically ill patients, particularly those with acute kidney injury, conventional creatinine-based estimated GFR equations may have important limitations and may not accurately reflect actual renal filtration. A strength of our study is the relatively large patient cohort, which enabled separate evaluation of subgroups. The collection of a large number of samples required considerable time, and some samples were stored for several years before analysis. This prolonged storage may have affected plasma BDNF levels and therefore represents a potential limitation of the study.

## 5. Conclusions

This study demonstrates that plasma BDNF levels are similarly reduced in patients with SIRS/sepsis/septic shock and are even lower in those with AKI-D. In contrast, patients with pancreatitis exhibited elevated plasma BDNF levels. Platelet count was associated with plasma BDNF levels in critically ill patients, and the clinical relevance of this association, particularly in patients with low or high platelet counts, warrants further investigation.

## Figures and Tables

**Figure 1:**
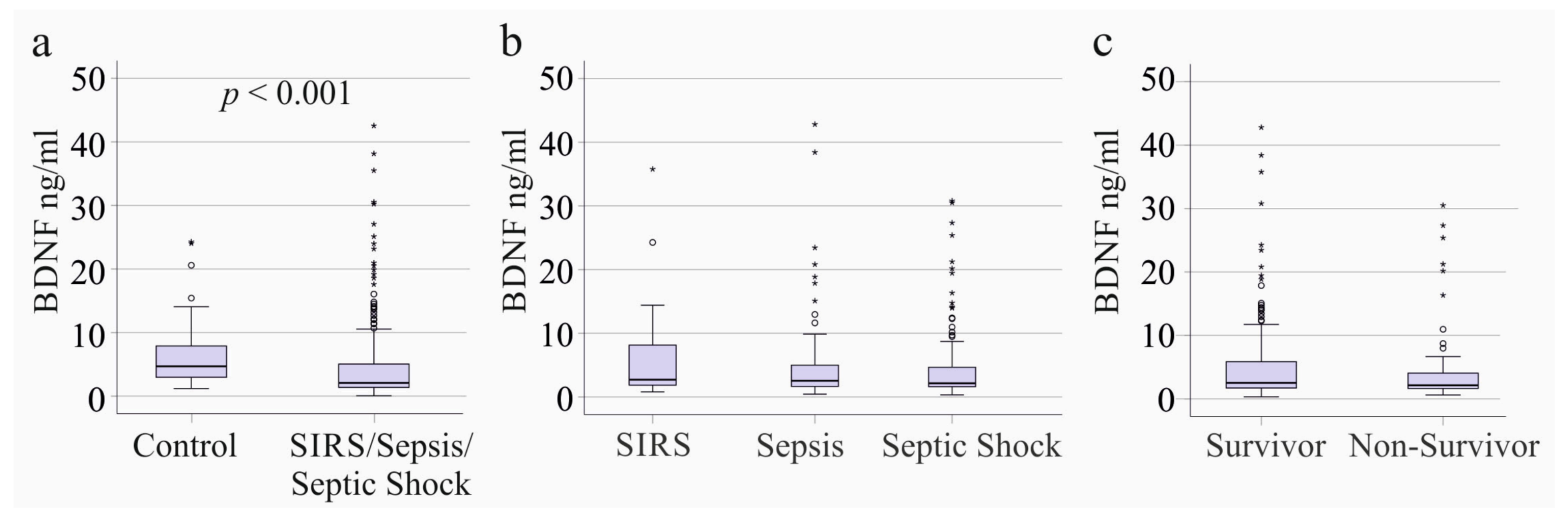
BDNF in plasma of controls and SIRS/sepsis/septic shock patients (57, 66 and 127 patients, respectively). (**a**) Plasma BDNF levels of the 40 controls and the 250 SIRS/sepsis/septic shock patients; (**b**) Plasma BDNF levels of patients with SIRS, sepsis, or septic shock; (**c**) Plasma BDNF levels of survivors and non-survivors (65 patients). Small circles and asterisks are outliers.

**Figure 2:**
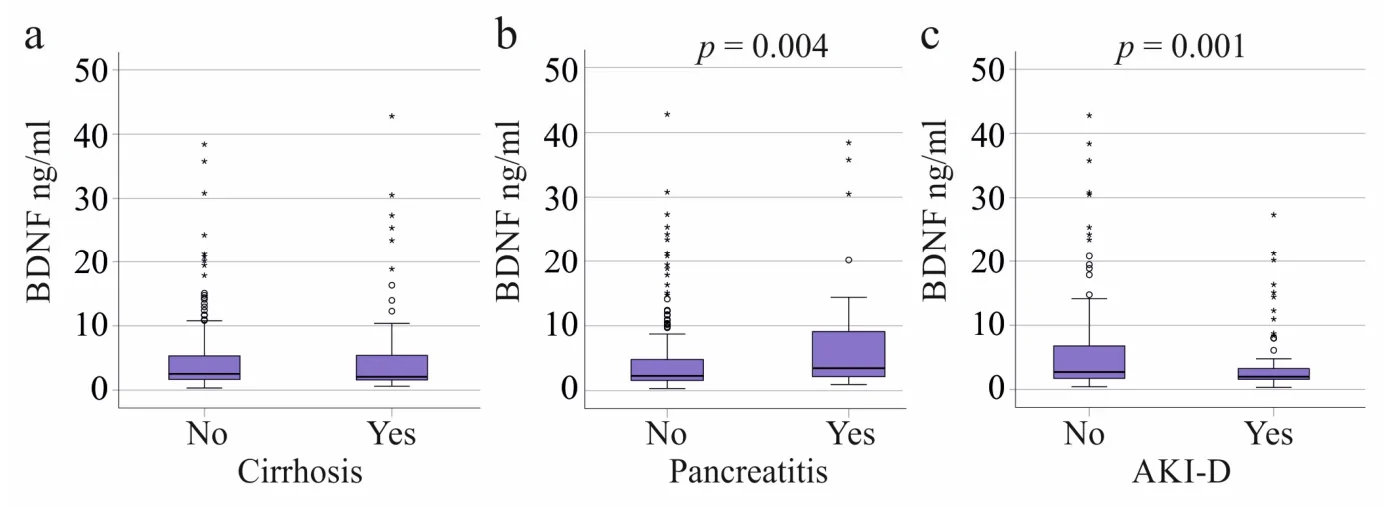
BDNF in plasma of SIRS/sepsis/septic shock patients in relation to underlying diseases and to dialysis. (**a**) Plasma BDNF levels of SIRS/sepsis/septic shock patients without (194 patients; No) and with (56 patients; Yes) cirrhosis; (**b**) Plasma BDNF levels of SIRS/sepsis/septic shock patients without (206 patients) and with (44 patients) pancreatitis; (**c**) Plasma BDNF levels of patients with SIRS/sepsis/septic shock without (156 patients) and with (94 patients) acute kidney injury requiring dialysis (AKI-D). Small circles and asterisks are outliers.

**Figure 3:**
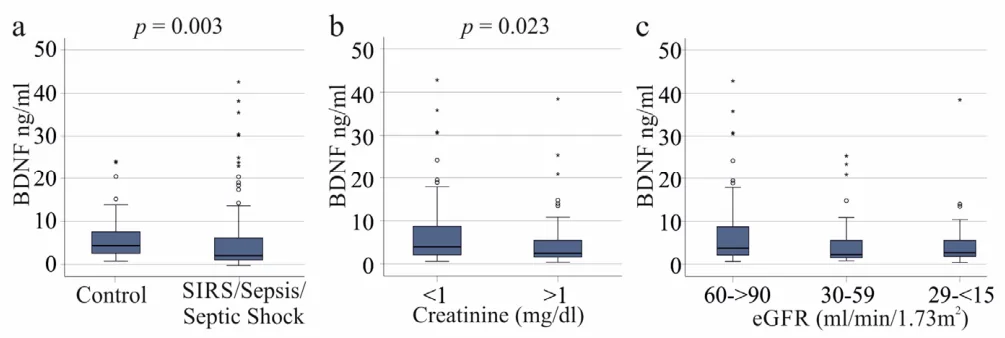
BDNF levels in plasma of controls and SIRS/sepsis/septic shock patients not on dialysis in relation to renal function. (**a**) Plasma BDNF levels of the 40 controls and the 156 SIRS/sepsis/septic shock patients not requiring dialysis; (**b**) Plasma BDNF levels of the SIRS/sepsis/septic shock patients with creatinine below (61 patients) and above (81 patients) 1 mg/dL; (**c**) Plasma BDNF levels of the SIRS/sepsis/septic shock patients stratified for estimated glomerular filtration rate (eGFR; 60–>90 mL/min/1.73 m^2^ (73 patients), 30–59 mL/min/1.73 m^2^ (44 patients), and 29 to <15 mL/min/1.73 m^2^ (28 patients). Small circles and asterisks are outliers.

**Figure 4:**
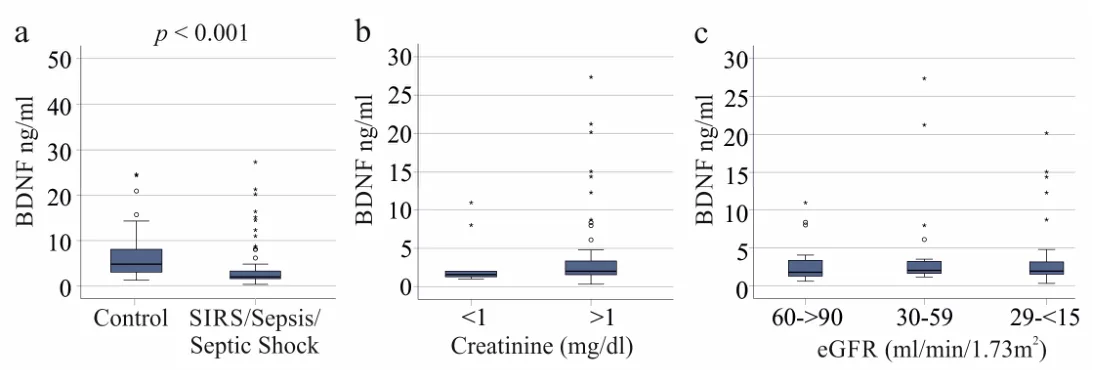
BDNF levels in plasma of controls and SIRS/sepsis/septic shock patients with AKI-D in relation to renal function. (**a**) Plasma BDNF levels of the 40 controls and the 94 SIRS/sepsis/septic shock patients requiring dialysis; (**b**) Plasma BDNF levels of the SIRS/sepsis/septic shock patients with creatinine below (10 patients) and above (71 patients) 1 mg/dL; (**c**) Plasma BDNF levels of the SIRS/sepsis/septic shock patients stratified by estimated glomerular filtration rate (eGFR; 60–>90 mL/min/1.73 m^2^ (18 patients), 30–59 mL/min/1.73 m^2^ (19 patients), and 29 to <15 mL/min/1.73 m^2^ (47 patients). Small circles and asterisks are outliers.

**Table 1:** Characteristics of SIRS/sepsis/septic shock patients without (−) and with (+) acute kidney injury requiring dialysis (AKI-D). Data are presented as medians, minimums, and maximums. The interquartile ranges (IQRs) are also given. Superscript numbers indicate the number of patients with available data when values were missing for some patients in the cohort. Empty cells in the *p*-value column denote non-significant differences (*p* ≥ 0.05). Statistical tests used: Mann–Whitney U test and chi-squared test.

Parameters	No AKI-D (156)	AKI-D (94)	*p*-Value
Males/Females	113/43	69/25	
Age (years)	61 (17–93); IQR = 23	59 (21–84); IQR = 16	
Body Mass Index (kg/m^2^)	26.6 (13.2–60.2); IQR = 7.5	27.8 (17.5–51.0); IQR = 12.00	
SIRS/Sepsis/Septic Shock	50/54/52	7/12/75	<0.001
C-reactive protein mg/L	138 (6–697); IQR = 174	132 (2–518); IQR = 145	
Procalcitonin ng/mL	1.40 (0.06–497.00); IQR = 7.51	3.64 (0.18–120.00); IQR = 6.59	0.004
Leukocytes n/nl	10.22 (0.28–246.94); IQR = 10.18	12.75 (0.54–57.91); IQR = 10.86	
Neutrophils n/nl	8.61 (0–72.59); IQR = 9.54	10.50 (0.46–50.61); IQR = 10.66	
Basophils n/nl	0.03 (0–3.20); IQR = 0.06	0.04 (0–0.52); IQR = 0.07	
Eosinophils n/nl	0.03 (0–8.80); IQR = 0.22	0.02 (0–0.94); IQR = 0.12	
Monocytes n/nl	0.65 (0–45.00); IQR = 0.92	0.76 (0.01–14.80); IQR = 1.14	
Lymphocytes n/nl	0.81 (0.01 –28.60); IQR = 0.86	0.72 (0 –3.82); IQR = 0.85	
Platelets n/mL	133 (4–794); IQR = 204 ^133^	80 (13–616); IQR = 112 ^80^	0.002
Immature Granulocytes n/nl	0.17 (0–6.19); IQR = 0.43	0.23 (0–7.25); IQR = 0.58	
Aspartate aminotransferase U/L	48 (7–1703); IQR= 63^146^	74 (13–8174); IQR = 127 ^91^	<0.001
Alanine aminotransferase U/L	32 (5–1139); IQR = 45 ^139^	45 (5–2360); IQR = 86 ^91^	
Albumin g/L	28.1 (15.9–61.3); IQR = 5.70^143^	28.0 (6.3–43.6); IQR = 8.90 ^91^	
Gamma glutamyltransferase U/L	122 (11–1923); IQR = 185.75 ^141^	126 (11–1280); IQR = 247 ^85^	
Total Bilirubin mg/dL	0.85 (0.20–33.90); IQR = 1.90 ^148^	2.40 (0.20–26.80); IQR = 6.88 ^92^	<0.001
Direct Bilirubin mg/dL	0.60 (0.10–22.50); IQR = 1.47 ^148^	1.80 (0.10–19.20); IQR = 4.88 ^89^	<0.001
Indirect Bilirubin mg/dL	0.30 (0.05–11.40); IQR = 0.55 ^138^	0.75 (0.07–8.10); IQR = 2.51 ^84^	<0.001
Urea mg/dL	57 (8–232); IQR = 76 ^156^	89 (8–347); IQR = 80 ^94^	<0.001
Creatinine mg/dL	1.1 (0.3–8.9); IQR = 1.10 ^142^	2.3 (0.3–16.1); IQR = 2.57 ^81^	<0.001
Glomerular filtration rate ml/min/1.73 m^2^	60 (5–174); IQR = 63 ^145^	28 (3–134); IQR = 35 ^84^	<0.001

**Table 2:** Spearman correlation coefficients for the association of plasma BDNF levels with clinical and laboratory parameters in patients without and with acute kidney injury requiring dialysis (AKI-D). *p*-values are reported only for statistically significant correlations. We did not correct *p*-values for multiple comparisons.

Parameters	Non-AKI-D			AKI-D		
	r	*p*-value	95% CI	r	*p*-value	95% CI
Age (years)	−0.042			−0.021		
Body Mass Index (kg/m^2^)	0.175	0.034	0.009–0.333	0.020		
C-reactive protein mg/L	0.103			0.083		
Procalcitonin ng/mL	−0.102			−0.080		
Leukocytes n/nl	0.023			0.196		
Neutrophils n/nl	0.061			0.161		
Basophils n/nl	0.021			0.084		
Eosinophils n/nl	0.067			−0.210	0.043	−0.402–0.001
Monocytes n/nl	0.026			0.013		
Lymphocytes n/nl	0.094			−0.086		
Immature Granulocytes n/nl	−0.135			0.026		
Platelets n/mL	0.282	<0.001	0.126–0.424	0.314	0.002	0.113–0.490
Urea mg/dL	−0.208	0.011	−0.361–0.044	0.255	0.015	0.046–0.443
Creatinine mg/dL	−0.167	0.048	−0.327–0.003	0.113		
Glomerular filtration rate ml/min/1.73 qm	0.138			−0.111		
Aspartate aminotransferase U/L	−0.026			0.144		
Alanine aminotransferase U/L	−0.004			0.126		
Albumin g/L	0.097			0.196		
Gamma glutamyltransferase U/L	0.077			0.110		
Total Bilirubin mg/dL	−0.212	0.010	−0.365–−0.047	0.099		
Direct Bilirubin mg/dL	−0.240	0.003	−0.391–−0.077	0.063		
Indirect Bilirubin mg/dL	−0.248	0.003	−0.403–−0.079	0.030		

## Data Availability

The original contributions presented in this study are included in the article. Further inquiries can be directed to the corresponding author.

## Abbreviations

The following abbreviations are used in this manuscript:

AKI-D	Acute kidney injury requiring dialysis
BDNF	Brain-derived neurotrophic factor
CI	Confidence interval
eGFR	Estimated glomerular filtration rate
ICU	Intensive care unit
SARS-CoV-2	Severe acute respiratory syndrome coronavirus 2
SIRS	Systemic inflammatory response syndrome
